# AI-Based Diagnostic Platform Capabilities With Lyme Disease as a Use Case: Integrative Exploration

**DOI:** 10.2196/87529

**Published:** 2026-07-13

**Authors:** Sarah P Maxwell, Connie L McNeely, Abdollah Zeraatpisheh, Chris Brooks, Claire Maxwell, Kevin C Thomas

**Affiliations:** 1 Public Health, Public Management, and Public Policy, School of Economic, Political and Policy Sciences, The University of Texas at Dallas, 800 W Cambell, Richardson, TX, 75080, United States, 1 972-883-6469; 2 Schar School of Policy and Government, George Mason University, Fairfax, VA, United States; 3 Public and Non-Profit Management, School of Economic, Political and Policy Sciences, The University of Texas at Dallas, Richardson, TX, United States; 4 School of Biological Sciences, University of Oklahoma, Norman, OK, United States; 5 O'Donnell School of Public Health, The University of Texas Southwestern Medical Center, Dallas, TX, United States; 6 Laboratory for Human Neurobiology, Chobanian & Avedisian School of Medicine, Boston University, Boston, MA, United States

**Keywords:** symptom checker, diagnostic accuracy, Lyme disease, serologic testing, clinical diagnosis

## Abstract

**Background:**

Lyme disease (LD) is the most common vector-borne disease in the United States. It is difficult to diagnose because it can mimic numerous other conditions, and testing protocols may not be sufficient. Although the Centers for Disease Control and Prevention (CDC) recommend a 2-tiered serologic testing approach for LD diagnosis, many patients are diagnosed clinically based on criteria such as the erythema migrans rash and, particularly when the rash is not present, various symptom patterns, exposure history, other information, and clinician observations. Against this backdrop, online symptom checkers, using artificial intelligence (AI) processing techniques, are increasingly used to obtain diagnostic information and resources. With LD as a use case, this research applied a modified capabilities approach to explore the relative effectiveness and utility of AI-based tools in application and comparison to serologically (CDC+) and clinically based diagnoses.

**Objective:**

The overarching goal of this research was to provide a baseline exploration of AI-assisted diagnostic tools relative to more traditional medical assessment approaches in detecting complex infectious diseases. To assess the potential diagnostic utility (DU) of online symptom checker platforms with LD as a use case, this study aimed to (1) evaluate platform performance in identifying LD across different diagnostic cohorts; (2) compare symptom patterns and severity distributions among online LD diagnoses; and (3) identify the most frequently co-occurring conditions potentially misclassified as LD or vice versa.

**Methods:**

Data were drawn from a limited structured survey of patients with confirmed or probable LD, including diagnostic pathways (CDC+ and/or clinical), symptom profiles and severity, treatment history, and time to diagnosis. These patient cases were then entered into 3 leading AI-based symptom checker platforms—MediFind, Isabel, and WebMD—to examine diagnostic performance. Descriptive analytics, logistic regressions, and postestimation analyses were used to identify patterns of DU and interaction effects among cohort type, symptom severity, and AI-based platforms. This survey-based research was not intended to serve as an experiment or clinical trial.

**Results:**

DU varied significantly across platforms and symptom severity thresholds. DU improved at higher symptom severity thresholds (≥3; *P*<.001) and was slightly higher among clinically diagnosed cohorts compared to CDC+ cohorts (*P*=.21). Marginal analyses revealed that clinically diagnosed respondents were more sensitive to changes in severity, but with different levels of platform consistency across conditions. Note that DU was analyzed principally for exploratory purposes.

**Conclusions:**

This research serves as a preliminary and directive step for expanded data collection and a larger, more comprehensive study. The findings suggest that AI-based symptom checkers may supplement early diagnostic reasoning in complex conditions such as LD, particularly when symptom severity is high. Inconsistency across platforms and diagnostic categories highlights the need for algorithmic refinement and standardized validation frameworks to enhance diagnostic reliability in AI-based tools.

## Introduction

### Background and Research Context

Lyme disease (LD) is the most common vector-borne disease in the United States, with recent estimates indicating increased numbers of new diagnoses and the treatment of approximately 476,000 individuals each year [1]. However, LD presents with a wide range of multisystem symptoms, and patients often face diagnostic difficulties. In fact, given these difficulties, there are some suggestions that the actual number of cases is grossly underestimated [2]. The perniciousness and expanding prevalence of LD—caused primarily by the bacterium *Borrelia burgdorferi* (and sometimes *Borrelia mayonii*) and spread to humans through the bite of infected blacklegged ticks—has been attributed to various factors, including environmental changes leading to wider tick breeding habitats.

At the same time, digitalization, large-scale data collection, and algorithmic engagement have become increasingly integral and expected in the health arena, not to mention across other domains. In particular, society has witnessed increasing developments in digital health resources available to the public (as well as to health professionals) and used as popular means for accessing relevant information, checking symptoms, and determining possible conditions and treatments. Of special note in this regard are symptom-checking applications, that is, online or software tools that provide a platform for patient and clinician use.

Symptom checker platforms allow users to input a variety of symptoms along with physical and other personal characteristics (age, sex, health history, geographic location, etc), which are then analyzed to produce different diagnoses, typically presented as a ranking in terms of diagnostic likelihood and potential seriousness [3-5]. Symptom checkers do not ask users for their medications, vital signs, or other questions that might otherwise be incorporated into a physical examination [4,6,7].

With artificial intelligence (AI) and natural language processing (NLP) as the basic means for analysis, symptom checker platforms typically require a brief medical and demographic history (eg, country of residence, sex, pregnant or not pregnant, and age). AI and NLP algorithms convert patient responses into different diagnoses, often listed in order of potential seriousness. Online symptom checkers use different AI capabilities, offering opportunities for improved diagnostic access and cost efficiency when patients can be diverted from the emergency room (ER) or quickly diagnosed [8,9]. However, performance varies substantially across platforms, reflecting differences in data scope, machine learning models, and linguistic input design.

Diagnostic utility (DU) remains the central issue, and the effectiveness of symptom checker platforms in this regard is a fundamental concern for patients, clinicians, and public health in general [9]. Given their growing adoption, greater reliance on symptom checker platforms for initial guidance and confirming information on LD and other tick-borne diseases can be expected relative to their increasing prevalence. This is particularly important given the variety of possible symptoms and conditions associated with LD and their overlap with other unrelated diseases. Moreover, it points to the issue addressed in this study—the DU of online symptom checker platforms relative to LD as a use case. To address this, a 3-fold interrelated capabilities approach is used: (1) it compares and assesses online symptom checker efficacy in diagnosing LD; (2) it provides a symptomatological comparison relative to online LD diagnoses; and (3) it compares symptoms, other conditions, and information relative to online non-LD diagnoses. Taken together, these comparisons provide for an encompassing analysis of diagnostic concurrence and online symptom checker applications for LD diagnoses.

### Research Focus

In today’s rapidly evolving digital landscape, the capabilities of online symptom checker platforms also are changing and expanding in response to AI-driven developments. Moreover, online symptom checkers are increasingly accessed—by laypersons, patients, and medical professionals—for diagnostic information and resources. In these days of “big data” and extended algorithmic application and engagement, they are, more than ever, being viewed as authoritative and reliable sources for decision-making and guidance [9]. However, to what extent do these platforms operate as consistent and reliable assessment bases? This issue is explored here using LD in the United States as a use case, especially given the complexities associated with its diagnosis and its growing incidence across the population and country.

After brief discussions of LD testing and of online symptom checker platform characteristics as background, details are provided on the research goals and the data and methods used for this study. The analysis is then presented, along with a discussion of the findings framed relative to questions of DU and AI-based diagnostic platform capabilities. The conclusion provides a summary and suggestions for practical application and further study.

### LD Testing and Presentation

LD is difficult to diagnose. A common assumption is that the defining indicator of LD is the tell-tale “bull’s-eye” erythema migrans (EM) rash and, especially in endemic areas, the EM rash alone is often used in clinical diagnoses [10]. However, in actuality, many people do not develop the EM rash, and, even when it is present, clinicians may misinterpret or overlook it as indicative of LD. Moreover, testing protocols may not be sufficient. Testing for LD typically relies on either serologic or clinical diagnostic approaches. In particular, the Centers for Disease Control and Prevention (CDC) in the United States recommends a 2-tiered serologic testing with a predetermined number of positive bands indicating LD, referred to as “CDC+.” One limitation of this approach is that, following an infection, antibodies can take weeks to generate, and “these tests have limited sensitivity during the early period of infection when people with EM typically seek medical evaluation” [11]. Serologic testing is often negative in early-stage LD. Even those who seek medical attention for the EM rash and receive antibiotics as treatment “may produce overall lower levels of *B. burgdorferi* antibodies, may only produce antibodies against a limited repertoire of *Borrelia* antigens, and may have impeded IgM-to-IgG isotype switching. This may lead to false-negative serologic results during the acute clinical phase when the EM is apparent and occasionally even during the convalescent (post-treatment) phase of illness” [11]. In fact, while CDC guidelines are based on serology, it is common for a patient not to produce antibodies. The resulting test may, therefore, produce a false negative when the test is performed too soon. False negatives may also occur when a patient is immunocompromised, has received early treatment, or has late-stage LD [12,13]. These limitations underscore the diagnostic uncertainty surrounding LD.

All in all, inconsistent symptom presentation and testing for LD are a particular concern, given the false negative and false positive occurrences. A lack of antibody production during testing can be problematic, leading some patients to seek out specialty laboratories for clinical testing. At least 3.4 million LD tests are performed by specialty laboratories in the United States each year [14,15]. Still, testing itself remains an issue of debate, especially since the Lyme bacterium is difficult to culture [16]. Moreover, LD is often called “the great imitator,” given its symptom similarities with other diseases and its multisystem nature, often affecting multiple organ and tissue systems. It is not unusual for such circumstances to lead to decision fatigue for clinicians [17]. Note, too, that misdiagnoses are more likely in urgent care and ER settings and tend to occur due to symptom misattribution, intermittent symptoms, and the belief of some physicians that an EM rash must be present [18]. Additionally, clinical LD diagnoses sometimes lead to further controversy, as clinicians may fail to test for related co-infections and other diseases with similar presentations [19]. Collectively, these challenges highlight why symptom-based and algorithmic approaches are increasingly considered in LD evaluation.

A final point is that patients diagnosed with LD often develop chronic or long-term symptoms. Lyme infection–associated chronic illnesses can occur after acquiring LD, often presenting as “debilitating physical symptoms including chronic fatigue, recurring pain, cognitive dysfunction such as ‘brain fog,’ and sleep disturbances despite antibiotic treatment for Lyme disease. Approximately 10‐20 percent of the 476,000 individuals who develop Lyme disease each year…go on to develop Lyme-IACI” [11]. Persistent postinfection symptoms pose significant challenges for patients and clinicians, underscoring the need for early and accurate diagnosis. Patients with LD often report a lower quality of life and greater health care usage than those with other chronic illnesses [20-22]. This study offers previously untested variables, using both serological and clinical diagnoses among patients with varying degrees of symptom severity prior to COVID-19.

### AI Symptom Checker Platforms

Symptom checker online platforms apply NLP, a subfield of AI, to analyze symptoms along with other possibly applicable information entered by the user. Platforms require typical background information such as a basic history of diagnoses, country of residence, and other demographic details, and NLP techniques and AI algorithms are applied to patient responses to determine possible diagnoses and health impacts. Regarding their general use, assessments of symptom checker accuracy against the final diagnosis by various medical providers—ranging from emergency department personnel to general and specialized residents and surgeons—have been highly variable [9]. Platforms also vary in their search features and analytical approaches. For example, studies have shown that some platforms use only 1 symptom (which can be expressed at various levels of detail, from general to fine-grained, and use various expressive descriptors and delineations), whereas others use as many as 20 for analysis [9].

Moreover, depending on the particular platforms and the medical conditions in question, the highest efficacy rate remains relatively low, but symptom checker platforms are continuously being updated and are advancing rapidly via AI and machine learning developments. Technological advances continue to improve algorithmic reasoning and language comprehension, although standardization across platforms remains limited. Currently, reported diagnostic accuracy rates for symptom checkers range from approximately 3% to 53%, depending on the condition set and model design [9].

However, symptom checkers have been used as useful tools in prescreening for diseases such as COVID-19 [23]. In a recent study of diagnostic accuracy of symptom checkers for COVID-19, only 2 of the 10 platforms balanced sensitivity (true positives) and specificity (patients without the disease), while most of the other checkers were particularly sensitive. Overly sensitive symptom checkers resulted in the classification of almost all patients as COVID-19–positive [23]. Other studies use comparisons with physician diagnoses rather than laboratory tests, noting that, in an emergency department setting, “the overall performance of Ada on its top 5 diagnoses compared with the ED diagnoses was not significantly different from that of the study physicians assessed on their top 3 diagnoses” [4]. Other studies indicate that diseases with atypical presentations or those that are less common tend to have lower diagnostic accuracy [24].

The NLP programs themselves can also have important effects on how the platforms are used and on the results they provide. NLP models have demonstrated capabilities for understanding and responding to human input [25], but outcomes can vary depending on particular programs and applications. For example, one widely used platform called Isabel uses more everyday or layperson “common” language [7], whereas others, such as the well-known WebMD, use more medically focused language [6,7,26-29]. Thus, the term “brain fog” does not appear as a symptom in WebMD; users would need to enter an alternative such as “altered mental state,” which may or may not occur to them or seem like an accurate description from their perspective. NLP has been applied to analyzing symptoms, such as depression, with a range of recognized conditions and outcomes. Accordingly, in this case, based on NLP, generative AI programs (eg, ChatGPT) have been used in determining and supporting the appropriate standard of care for patients with both mild and severe depression [30]. These applications illustrate both the promise and limitations of AI-assisted health technologies in translating subjective experiences into diagnostically meaningful data.

## Methods

### Study Design and Data Sources

This study draws on the extensive review conducted by Wallace et al [9] on the diagnostic and triage accuracy of digital and online symptom checker tools as a foundation for examining their utility in relation to LD. Diagnostic determinants in this regard are based primarily on symptoms [29], patient entry point (eg, ER vs non-ER) [7], or physician specialty [27]. This research also incorporates multiple symptom checker applications with more common-language and medical-language programs, which may vary across platforms. Focusing on patients with LD, it more specifically compares individuals with serologically diagnosed (CDC+) versus clinically diagnosed LD based on tests performed in commercial laboratories.

Individual patient data were drawn from an online survey conducted in 2019 (prior to the onset of the COVID-19 pandemic), disseminated via Facebook and shared among various LD-affiliated organizations and individuals. This resulted in a convenience sample of 67 participants. We recognize that this recruitment strategy, which relied on capturing patients from various LD groups, can introduce selection bias into the sample. For example, it is conceivable that this approach favors patients with chronic, atypical, or polysymptomatic presentations. However, as an exploratory, initializing, and directive step, this approach still offers a fundamental contribution to the literature, as previous symptom checker studies tended to focus on definitive diagnoses rather than conditions that are difficult to diagnose.

Patients who self-reported an LD diagnosis were asked to document their diagnostic type (CDC+ and/or clinical). CDC+ was defined for the respondents using the National Notifiable Diseases Surveillance System [31]. Patients were asked to report their symptoms and symptom severity, as well as other diagnostic-related and treatment-related information, including the time taken to receive a diagnosis and the related financial costs. As shown in Textbox 1, a wide range of LD symptoms has been identified by the CDC and in the medical literature [32], accounting for much of the associated diagnostic difficulty. Additionally, some language or terms used by patients (eg, “brain fog”) do not appear explicitly as CDC-recognized symptoms. This research endeavored to include such symptoms as reported by patients for a more comprehensive symptom delineation and recognition.

Textbox 1. **Identified Lyme disease symptoms.**

**Centers for Disease Control and Prevention (CDC)–recognized symptoms**

- Rash
- Fever or chills
- Fatigue
- Malaise
- Headache
- Myalgia
- Arthralgia
- Pharyngeal erythema
- Lymphadenopathy
- Baker cyst
- Conduction abnormalities
- Myocarditis or pericarditis
- Bell palsy or other cranial neuropathy
- Meningitis
- Motor and sensory radiculoneuropathy and mononeuritis multiplex
- Subtle cognitive difficulties
- Encephalitis, encephalomyelitis, subtle encephalopathy, and pseudotumor cerebri

**Additional symptoms from the medical literature**

- Confusion or altered mental status
- Pain
- Seizures
- Vertigo or dizziness (not reported by CDC)
- Tingling or numbness (not reported by CDC)
- Impaired cognitive function (concentration, memory difficulty, and word recall; not reported by CDC)
- Paralysis: difficulty swallowing (dysphagia) or Bell palsy
- Difficulty with, or slurred speech (dysarthria; not reported by CDC)
- Fainting (syncope; not reported by CDC)
- Depression
- Anxiety (not reported by CDC)
- Mania, panic attacks, delusions, or hallucinations (not reported by CDC)

During July and August 2023, patient-reported symptoms and other relevant information were entered into 3 online symptom checker platforms—MediFind, Isabel, and WebMD. These platforms were selected based on their high levels of recognition, frequency of use, variability in approach, and reported utility [33]. Respondent symptoms were entered according to severity and diagnostic type (clinical or CDC+). MediFind is an online medical website providing medical information and a symptom checker tool designed to identify diseases from peer-reviewed journals, clinical trials, and medical presentations. It is based on NLP and a wide-scoping AI algorithm formation. The MediFind website indicates that its proprietary algorithms use the simplified terminology, allowing users to enter symptoms in “natural” or common language, as well as more medically grounded language [34]. The Isabel symptom checker website states that it is “recognized worldwide for its utility covering both common and rare conditions” [33], suggesting potential in relation to difficult-to-diagnose diseases such as LD.

In 2018, WebMD launched upgraded technology that allows users to enter “easy-to-understand” language into the platform, although levels of applicability and understanding could vary substantially by user. However, compared with other platforms, WebMD was more limited in language and expression and did not offer common-language options for many symptoms. The following changes and observations were made while entering symptoms into WebMD, given the more technical medical language restrictions of the program. These substitutions were applied consistently to ensure cross-platform comparability of input terms. All symptoms were those listed by the respondents. The change to medically indicated language (eg, “brain fog” to “altered state of mind”) was entered based on the occurrence of the only comparable symptoms from which to select such that the medical language was standardized via the online platform.

- “Intermittent pain” and “shooting pains,” as described by patients, were not recognized by WebMD. These symptoms were replaced with “pain.”
- “GI or stomach problems” was replaced with “nausea.”
- “Brain fog” was replaced with “altered state of mind.”
- WebMD did not have an option for “panic attacks.” However, “anxiety” in general was an included symptom.
- “Extreme fatigue” was substituted with “fatigue.”

As mentioned earlier, data entry for this study took place in 2023. At that time, none of the symptom checkers allowed input on symptom severity. Thus, symptom severity was split for each individual respondent into ≤2, which was a natural distinction in minor or more severe symptom presentation, and ≥3; these are actually nested in that ≥3 includes the initial ≤2. Symptom severity for each symptom was entered as follows: first, all symptoms with a severity score of ≥3 were entered, and then, keeping the most severe symptoms in the symptom checker program, the remaining reported symptoms were added for each respondent with a severity ≤2 to the original list. Two sets of diagnoses were produced by each symptom checker platform: one for the most severe symptoms only, and a second set of diagnoses for a respondent’s full list of symptoms. Finally, the top 10 diagnoses were obtained by severity and by clinical or CDC+ diagnoses. (If “COVID” resulted as a symptom checker output diagnosis, it was removed.)

In general, analyses were conducted on a “Lyme Disease Artificial Intelligence Diagnosis” dataset constructed for this study, as described above. Constructing the raw LD and AI dataset included unifying the 3 subdatasets (one for each AI platform), cleaning and standardizing the data, and encoding it numerically to facilitate analysis. This dataset consists of self-reported symptoms and AI-generated diagnoses, with the main analysis focused on characterizing potential differences in DU across 3 major categorical variables—cohort, symptom severity threshold, and AI platform. These variables are defined in the following sections.

### Dependent Variable

The dependent variable examined in this study was DU.

- DU is determined by the proportion of principal diagnoses that include LD. It is dichotomous, indicating values of “No” and “Yes.” It is the proportion of these values across defined groups.

### Independent Variables

Three independent variables were included in the analyses.

- *Cohort* defines which LD diagnostic cohort was input into the diagnostic AI platform. It has 2 values: “CDC+” (those diagnosed using CDC serology testing) and “Clinical” (those diagnosed using clinical testing).
- *Symptom severity threshold* (SSTCat) is based on each threshold determined by the number of symptoms as entered into the diagnostic AI platforms. It has 2 values: “Less than or equal to 2” (LT2,≤2) and “Greater than or equal to 3” (GT3,≥3).
- *AI platform* defines which AI-based symptom checker provided the diagnoses. It has 3 values: “MediFind” (MF), “Isabel” (IS), and “WebMD” (MD).

In total, there are 2 × 2 × 3=12 subcategories definable by these categorical variables (CatVars), each with its own set of DU data.

Analysis was undertaken to determine the potential effects that may influence the ability of an AI-based symptom checker to accurately diagnose an individual with LD. In addition to descriptive analytics, logistic regressions were performed to assess the association among variables and to classify and predict associated outcomes. Moreover, postestimation analysis was used for follow-up validity checks on platform comparisons and to identify the principal effects. Analyses were aimed at (1) assessing and comparing the DU of the 3 online diagnostic tools, (2) evaluating diagnostic concurrence with symptoms and test type relative to AI-based outcomes, and (3) comparing variance in LD as opposed to non-LD diagnoses given matching symptom and other information input across platforms. Collectively, these analyses were used to evaluate both platform-specific and cross-cohort diagnostic consistency.

### Ethical Considerations

Institutional review board approval (IRB-21-149) for the study protocol was obtained prior to the survey under the formal adoption of the Declaration of Helsinki. All responses were completely anonymous, and survey respondents consented to voluntarily participate in the survey. All survey respondents provided informed consent. No personally associated identification data were collected or reported as such. Participants were not compensated. Data are available upon request.

## Results

All sample respondents had an LD diagnosis. A total of 65 participants constituted the initial study sample, of which 39 were in the CDC+ diagnosis cohort and 26 were in the clinical diagnosis cohort. The study sample consisted of 58 (89.2%) female participants and 7 (10.8%) male participants, with an average age of 41.8 (SD 15.3) years (range: 8‐73). The CDC+ cohort consisted of 34 (87.2%) females and 5 (12.8%) males, with an average age of 40.4 (SD 16.5) years (range: 8‐73). The clinical cohort consisted of 24 (92.3%) females and 2 (7.7%) males, with an average age of 44 (SD 13.4) years (range: 19‐72).

**Figure 1.**
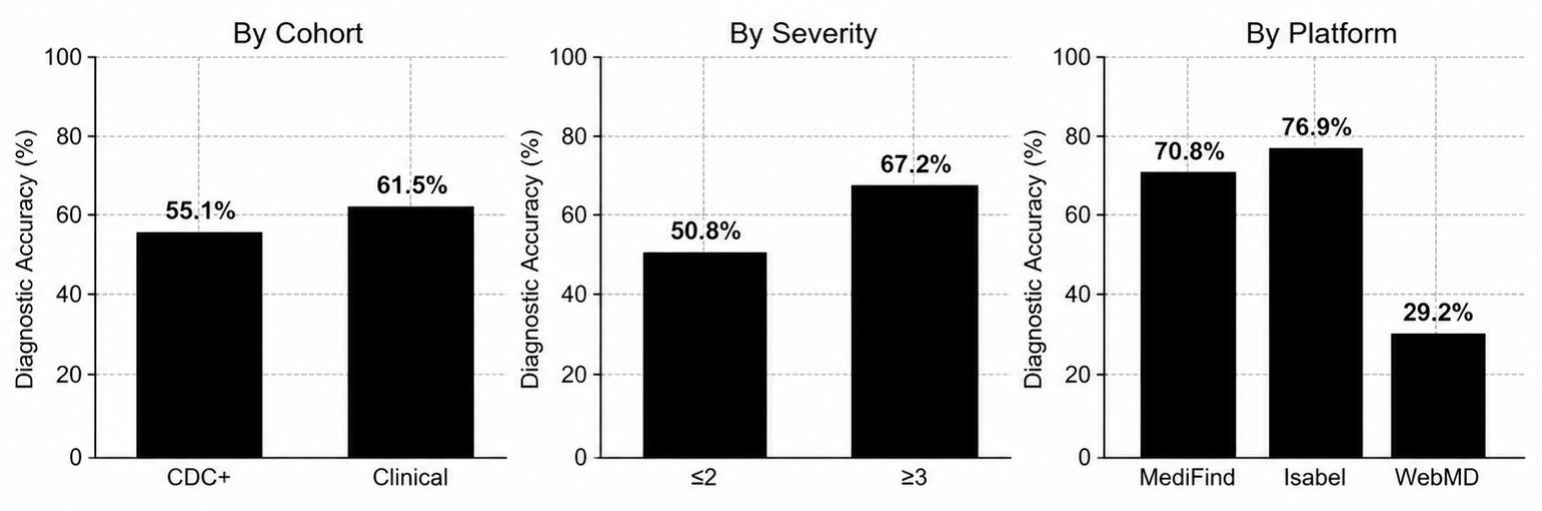
Diagnostic utility relationships among cohort type, symptom severity thresholds, and artificial intelligence platform.

**Figure 2.**
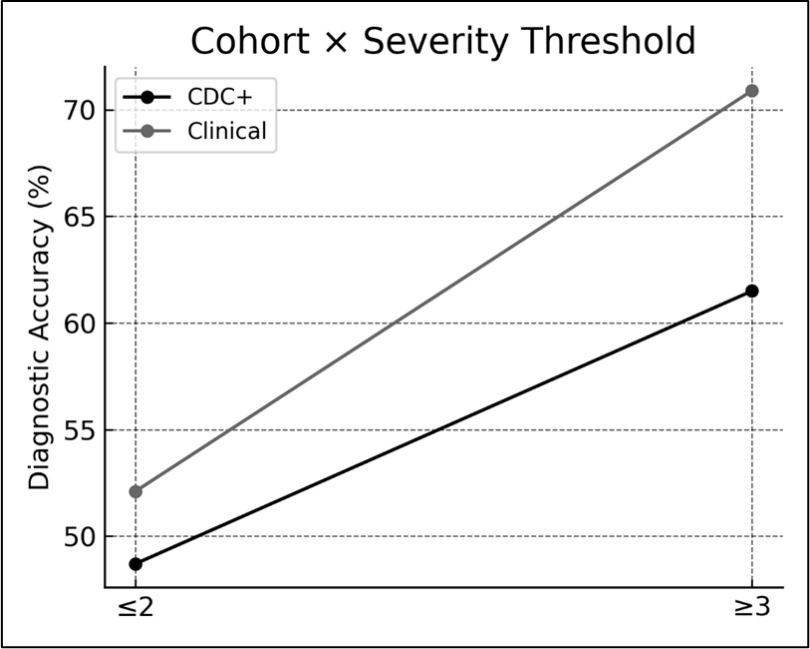
Frequency of Lyme disease diagnoses by cohort and symptom severity.

Given this sample, a comparative baseline for evaluating AI-driven diagnostic performance in LD across diagnostic categories was derived relative to the research framework, as shown in Figure 1, which summarizes the diagnostic distributions and categorical variable structure forming the basis for the overall analysis. Moreover, these symptom patterns identified in the survey informed the baseline comparison of diagnostic outputs, as visually summarized in Figure 2.

Given the research aims for this study, the following questions were posed to guide the analysis: (1) “Does DU significantly vary within the 3 categorical variables (CatVars)?” and (2) “Are there any interactions between categorical variables (CatVars) that significantly influence DU?” (Null hypothesis: DU does not significantly vary within or between the CatVars). The main objectives were to compare and assess similarities and differences between those with clinical versus CDC+ diagnoses and to determine the potential capabilities and efficacy of online symptom checkers relative to their potential for and levels of DU. To address these, 6 logistic regression models were fit: 3 univariate models for each individual categorical variable and 3 bivariate models for each possible pairing of the categorical variables. Odds ratios, *z* values, and *P* values, marginal estimates, and pairwise comparisons were extracted from each model (see Table S1 in Multimedia Appendix 1 for complete listing of model outcomes and comparisons).

**Figure 3.**
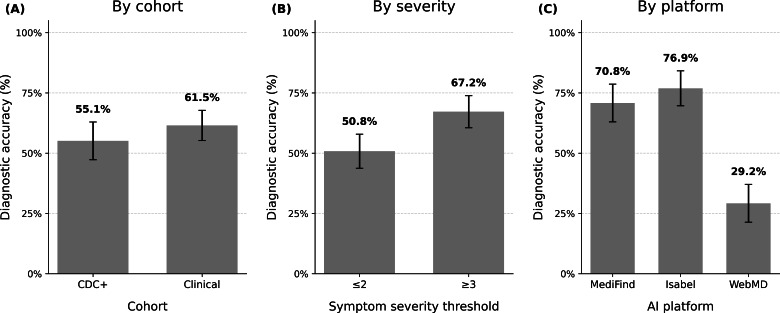
Univariate model results—diagnostic utility by artificial intelligence (AI) platform, cohort, and symptom severity threshold.

The univariate models each analyzed differences within a single categorical variable, without accounting for the other 2 categorical variables. Figure 3 illustrates the marginal DU estimates (with 95% CIs) by cohort, symptom severity threshold, and AI platform. DU did not vary significantly by cohort (*P*=.21), was significantly higher when the symptom severity threshold was greater than 3 (*P*<.001), and was significantly lower in WebMD (*P*<.001) relative to the other symptom checkers. MediFind and Isabel were not significantly different from each other (*P*=.26). Differences in DU could be due to not just database access, but also the design of the AI algorithms used and NLP learning effects, which may have implications for the capabilities of any AI-based symptom checker.

**Figure 4.**
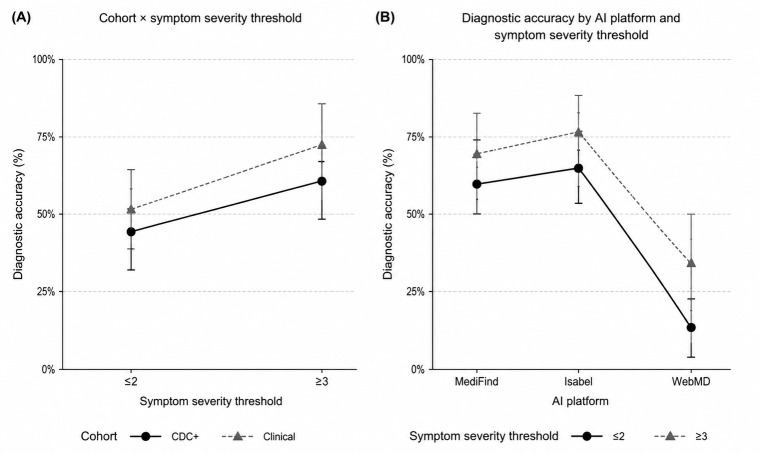
Bivariate model results—interaction effects between cohort type and symptom severity threshold: (A) cohort by symptom severity threshold and (B) symptom severity threshold by AI platform. AI: artificial intelligence; CDC: Centers for Disease Control and Prevention.

The bivariate models analyzed interactions between given pairs of categorical variables and included the main effects for each variable as well as the interaction effect between them. No significant interaction effects were observed in the base models (*P*>.12). Marginal estimates—predictions of the true value of DU in different combinations of the categorical variables—also were generated and evaluated. Several potential interaction effects were observed and subsequent pairwise comparisons were conducted to determine the magnitude and significance of these differences. Postestimation analyses also found a number of significant interactions. Figure 4 shows the interaction effects with 95% CIs, where Figure 4A shows cohort by symptom severity threshold, and Figure 4B shows symptom severity threshold by AI platform. Overall, regarding interaction effects across the AI platforms and cohorts, significantly higher DU was found with the ≥3 threshold than the ≤2 symptom severity threshold, but only in the clinically diagnosed LD cohort (*P*<.001). While there was no significant difference between the ≤2 and ≥3 symptom severity thresholds within the CDC+ cohort (*P*=.12), the difference became significant within the clinical cohort (*P*=.003), suggesting that the clinically diagnosed cohort may be more sensitive to changes in symptom severity. More specifically, the clinically diagnosed cohort received significantly higher DU than the CDC+ group, but only on the Isabel platform (*P*=.09). Isabel and MediFind had significantly higher DU, but only with the ≥3 symptom severity threshold (*P*[IS]=.04; *P*[MD]=.008). It may be that the different trends between the test platforms are canceling each other out, obfuscating interaction effects between diagnostic cohort and the AI applications, a point that merits further investigation and specification relative to AI-based diagnostic capabilities.

Note that the significant differences between the ≤2 and ≥3 symptom severity thresholds observed in the univariate models were nonsignificant in the bivariate models (*P*>.11). The largest and most consistent difference in DU was observed between the WebMD and the MediFind or Isabel platforms. While MediFind and Isabel had similar DUs, that of WebMD was significantly lower. Although the ≥3 symptom severity threshold may have significantly higher effects than ≤2, especially when restricted to the clinically diagnosed LD cohort, some of the difference appears to be due to interactions with the particular AI-based platform and cohort, as indicated by the ≤2 and ≥3 interaction main effect becoming nonsignificant in the bivariate models. The clinically diagnosed LD group was notionally higher than the CDC+ group, but this only became significant when restricted to the Isabel AI data. In general, both the MediFind and Isabel online symptom checkers varied significantly between the ≤2 and ≥3 symptom severity thresholds, suggesting an interaction. While there is some level of interaction between AI platforms and symptom severity threshold, greater DU is found with ≥3 than ≤2; however, MediFind appears to benefit in this case less than Isabel or WebMD. This may be an artifact due to MediFind having the highest baseline DU among them, suggesting the need for additional research controlling for such points.

## Discussion

### Principal Findings

The main objective of this study was to explore AI-assisted diagnostic tools relative to more traditional medical assessment approaches in detecting complex infectious diseases. The focus was on the potential DU of online symptom checker platforms with LD as a use case. A separate goal was to explore nuanced difficulties and contrasts with AI symptom checker input (eg, terminological differences and effects), which shed light on the lack of standardization among various AI platforms. The functionality of the AI platforms introduced unexpected complexities to the study, indicating the need for further research and exploration, particularly as AI develops and its use in medical settings expands.

However, even given the inherent limitations of the platforms, the potential for DU and detection is encouraging. Based on the logistic regressions, along with postestimation, testing for robustness and considering relative influences on the univariate and bivariate models, the major outcomes included a significantly lower DU on the WebMD platform—which could be due principally to differences in AI development and application—and a potentially higher DU in the ≥3 symptom severity threshold. No significant main effects were observed between the cohorts. Interactions among the categorical variables were at times significant, although it is unclear if they were spurious or evidence of a weak effect. This is something that should be further explored in future analyses. In any case, together, the findings underscore the value of comparing AI-based symptom checker performance through a multivariate framework and inform the broader discussion of AI diagnostic reliability in complex conditions such as LD.

The findings here highlight the point that, while DU varies across AI symptom checkers, these tools show measurable promise in differentiating cases of LD under specific symptom severity conditions. AI-based platforms may have the potential to aid in improved diagnostics for conditions and diseases such as LD for which diagnosis is notably challenging. Different from most studies that focus principally on results from clinician differential diagnosis or imaging, this research also incorporates reported serological findings for a more comprehensive perspective and analysis. To that end, a goal of this research was to determine and examine the fundamental associations between the categorical groups and the primary outcomes in order to inform future analysis and the role of, and reliance on, AI diagnostic platforms. Along those lines, this study contributes to the AI-diagnostic knowledge base and also suggests promise for individuals with lingering symptoms and no definitive diagnosis, as is often the case with LD.

### Limitations

Further research is needed with an eye toward conducting a more methodologically exacting assessment of the relevant issues. While this study serves as a preliminary directive exploration focused on the sensitivity of symptom checkers, it did not include a control group for comparison with participants without an LD diagnosis. Both sensitivity, which is the true-positive rate, and specificity, which can identify true negatives, can be problematic in survey designs, especially given the reliance on patient self-reported data. As mentioned, the sample recruitment approach also presented potential limitations, as it may favor patients with chronic, atypical, or polysymptomatic presentations. Note that regarding actual diagnoses and data quality, the survey responses might reflect a certain degree of recall bias, particularly in relation to symptom severity reported for past events, potentially influencing the correlation between reported severity and DU. In addition, the internal validity of the study could be compromised since the definitions used for clinical diagnoses may conflict with or undermine differing AI platform informational approaches relative to potentially unconfirmed diagnoses. Moreover, this points to AI developments and changing capabilities as a critical consideration affecting the potential feasibility and utility of symptom checker platforms over time. However, as noted, this study was exploratory and directive in purpose.

### Conclusions

AI diagnostic outputs depend not only on input structure but also on algorithmic design and NLP interpretation, indicating that performance gaps among platforms may reflect both data and model architecture. Attending to these kinds of issues is essential for refining AI-driven diagnostic frameworks and improving their reliability in relation to complex, multisystemic conditions such as LD. As indicated in the analysis, AI platform and symptom severity threshold appear to be the most influential of the categorical variables. Accordingly, future studies should generally account for them in the models that they develop. It may also be appropriate to account for diagnostic type and cohort in some models, as was done here. Moreover, although not pursued in this initial analysis, consideration of secondary variables, as mentioned, may provide a finer-grained understanding of related issues. Categorizing symptoms and diagnoses into several broad subgroups could allow for more granular analyses. Doing so would be trivial for symptoms that have predetermined groups (eg, physical, neurological), whereas diagnoses would require defining a categorization system and then applying it uniformly across different AI platforms, each of which could have its own list of unique diagnoses. In addition to data verification and the use of demographic data to account for intersubjective variability, directly incorporating details on symptoms and non-LD diagnoses can introduce and help to address further delineating questions. For example, what specific symptoms are most associated with successfully reaching an LD diagnosis (or not)? Especially in light of its mimicry character, which diagnoses are most often made in lieu of LD (ie, for which conditions is LD typically mistaken)? Which diagnoses are most often made alongside LD (ie, with which conditions is LD most often associated)? Such questions can be more directly addressed in light of different diagnostic capabilities associated with AI sources and algorithmic developments.

## Supplementary material

10.2196/87529 Multimedia Appendix 1 Summary analyses of marginal estimates and main and interaction effects.

## Abbreviations

AI: artificial intelligence
CDC: Centers for Disease Control and Prevention
DU: diagnostic utility
EM: erythema migrans
ER: emergency room
LD: Lyme disease
NLP: natural language processing
