# Supplementary material for: AI-Based Diagnostic Platform Capabilities With Lyme Disease as a Use Case: Integrative Exploration
Source: Online J Public Health Inform. 2026 Jul 13;18:e87529. doi: 10.2196/87529 (PMC13361615; doi:10.2196/87529)
Supplement: Multimedia Appendix 1 [file ojphi-v18-e87529-s001.docx]

**APPENDIX**

**Table A-1**: Summary Analyses (Marginal Estimates by Groups, and including outcomes of Main and Interaction Effects tests. Comparisons for Interaction Effects involve groups defined by two variables, as indicated by ‘#’ connecting them.)

| **Model** | **Group** | **Marginal Estimate**  **[Standard Error]** | **Marginal Estimate**  **[Standard Error]** *Note: Converted to %* | **Comparison: z-value (p-value)** *Note: Only Main Effects and Interactions shown Note: * < 0.10, ** < 0.05* |
| --- | --- | --- | --- | --- |
| Univariate, Cohort | CDC+ | 0.551 [0.040] | 55.1% [4%] | Clinical x CDC: 1.26 (0.208) |
| Univariate, Cohort | Clinical | 0.615 [0.032] | 61.5% [3.2%] |  |
| Univariate, Symptom Severity Threshold | LT2 | 0.508 [0.036] | 50.8% [3.6%] | ** GT3 x LT2: 3.28 (0.001) |
| Univariate, Symptom Severity Threshold | GT3 | 0.672 [ 0.034] | 67.2% [3%] |  |
| Univariate, AI | MF | 0.708 [0.040] | 70.8% [4%] | IS x MF: 1.13 (0.260) ** MD x MF: -6.48 (<0.001) ** MD x IS: -7.36 (<0.001) |
| Univariate, AI | IS | 0.769 [0.037] | 76.9% [3.7%] |  |
| Univariate, AI | MD | 0.292 [0.040] | 29.2% [4%] |  |
| Bivariate, Cohort x Symptom Severity Threshold | CDC#LT2 | 0.487 [0.057] | 48.7% [5.7%] | Clinical x CDC: 0.47 (0.640) GT3 x LT2: 1.61 (0.108) Clinical#GT3 x CDC#LT2: 0.67 (0.502) |
| Bivariate, Cohort x Symptom Severity Threshold | CDC#GT3 | 0.615 [0.055] | 61.5% [5.5%] |  |
| Bivariate, Cohort x Symptom Severity Threshold | Clinical#LT2 | 0.521 [0.046] | 52.1% [4.6%] |  |
| Bivariate, Cohort x Symptom Severity Threshold | Clinical#GT3 | 0.709 [0.042] | 70.9% [4.2%] |  |
| Bivariate, Cohort x AI | CDC#MF | 0.635 [0.067] | 63.5% [6.7%] | Clinical x CDC: 1.49 (0.137) IS x MF: 0.62 (0.534) ** MD x MF: -3.09 (0.002) ** MD x IS: -7.05 (<0.001) Clinical#IS x CDC#MF: 0.22 (0.824) Clinical#MD x CDC#MF: -1.55 (0.121) |
| Bivariate, Cohort x AI | CDC#IS | 0.692 [0.064] | 69.2% [6.4%] |  |
| Bivariate, Cohort x AI | CDC#MD | 0.327 [0.065] | 32.7% [6.5%] |  |
| Bivariate, Cohort x AI | Clinical#MF | 0.756 [0.049] | 75.6% [4.9%] |  |
| Bivariate, Cohort x AI | Clinical#IS | 0.821 [0.043] | 82.1% [4.3%] |  |
| Bivariate, Cohort x AI | Clinical#MD | 0.269 [0.050] | 26.9% [5%] |  |
| Bivariate, Symptom Severity Threshold x AI | LT2#MF | 0.646 [0.059] | 64.6% [5.9%] | GT3 x LT2: 1.53 (0.125) IS x MF: 0.56 (0.576) ** MD x MF: -5.07 (<0.001) ** MD x IS: -7.38 (<0.001) GT3#IS x LT2#MF: 0.50 (0.619) GT3#MD x LT2#MF: 0.84 (0.398) |
| Bivariate, Symptom Severity Threshold x AI | LT2#IS | 0.692 [0.057] | 69.2% [5.7%] |  |
| Bivariate, Symptom Severity Threshold x AI | LT2#MD | 0.185 [0.048] | 18.5% [4.8%] |  |
| Bivariate, Symptom Severity Threshold x AI | GT3#MF | 0.769 [0.052] | 76.9% [5.2%] |  |
| Bivariate, Symptom Severity Threshold x AI | GT3#IS | 0.846 [0.045] | 84.6% [4.5%] |  |
| Bivariate, Symptom Severity Threshold x AI | GT3#MD | 0.400 [0.061] | 40% [6.1%] |  |
